# Use of light-weight foaming polylactic acid as a lung-equivalent material in 3D printed phantoms

**DOI:** 10.1007/s13246-023-01318-4

**Published:** 2023-09-06

**Authors:** Scott Crowe, Sarah Maxwell, Harsimran Brar, Liting Yu, Tanya Kairn

**Affiliations:** 1 Cancer Care Services, Royal Brisbane & Women’s Hospital, Brisbane, QLD Australia; 2 Herston Biofabrication Institute, Brisbane, QLD Australia; 3 School of Information Technology and Electrical Engineering, University of Queensland, Brisbane, QLD Australia; 4 School of Chemistry and Physics, Queensland University of Technology, Brisbane, QLD Australia

**Keywords:** 3D printing, Radiotherapy, Phantoms, Lung

## Abstract

The 3D printing of lung-equivalent phantoms using conventional polylactic acid (PLA) filaments requires the use of low in-fill printing densities, which can produce substantial density heterogeneities from the air gaps within the resulting prints. Light-weight foaming PLA filaments produce microscopic air bubbles when heated to 3D printing temperatures. In this study, the expansion of foaming PLA filament was characterised for two 3D printers with different nozzle diameters, in order to optimise the printing flow rates required to achieve a low density print when printed at 100% in-fill printing density, without noticeable internal air gaps. Effective densities as low as 0.28 g cm^− 3^ were shown to be achievable with only microscopic air gaps. Light-weight foaming PLA filaments are a cost-effective method for achieving homogeneous lung-equivalency in 3D printed phantoms for use in radiotherapy imaging and dosimetry, featuring smaller air gaps than required to achieve low densities with conventional PLA filaments.

## Introduction

Additive manufacturing, or 3D printing, has allowed the fabrication of a wide variety of jigs and tissue-mimicking phantoms in medical physics [1]. These bespoke phantoms and modular components can be used to assess the performance of other devices (such as imaging and radiation therapy treatment systems) at a low cost [2] and can extend the functionality of commercial solutions. The ability to make modular anthropomorphic and even individualised phantoms, containing lung and bone mimicking materials, is useful when commissioning new equipment and techniques.

Many 3D printable materials have mass and electron densities equal to or exceeding those of water and soft tissues. For example, polylactic acid (PLA) has a nominal density of 1.3 g cm^− 3^. To achieve radiological properties equivalent to a desired tissue within a phantom, the in-fill density of the printed material can be varied. For lung, for example, in-fill density needs to be reduced to achieve densities between 0.2 and 0.45 g cm^− 3^ [3, 4]. The in-fill density is the ratio of printed material compared to air within in a unit volume, and it can be optimised to achieve a desired effective radiological density using CT imaging, or physical or transmission measurements of printed samples for the printer and material being used [3, 5]. To minimise variations in the path length in the printed material for radiation that is incident from different angles (i.e., to achieve an isotropic radiological density), this in-fill density should be achieved using a gyroid in-fill pattern [6].

The use of varied in-fill density has been used to mimic lung tissue in numerous 3D printed phantoms described in the literature for use in radiology and radiation therapy [1, 7–9]. However, the presence of substantial density heterogeneities for very low in-fill densities may introduce uncertainty in measurements performed in small radiotherapy fields [10]. These air gaps can be reduced through the use of low-density filaments, such as Woodfill (colorFabb, Belfeld, Netherlands), a composite of PLA and wood fibres [3]. Polymer foams and commercially available phantom materials, such as Gammex LN-300 and LN-450 (Gammex Inc, Middleton, USA) and CIRS inhale and exhale lung (CIRS Inc., Norfolk, USA) achieve lung equivalence without large air gaps, and more closely resemble lung tissue featuring microscopic air-filled alveoli, reducing this uncertainty.

Light-weight foaming PLA is a 3D-printable filament containing a chemical foaming agent that decomposes and releases gas when exposed to filament extrusion temperatures used in fused deposition modelling 3D printing [11]. This decomposition results in the creation of microscopic bubbles (less than 0.1 mm in diameter) within the extruded PLA, increasing the printed filament diameter relative to the diameter of the 3D printing extrusion nozzle. The size and quantity of bubbles, and therefore density of the material, varies with printing temperature and extrusion flow rate [11].

The objective of this study was to characterise the use of 3D-printable light-weight foaming PLA as a lung-mimicking material for use in radiology and radiation therapy phantoms.

## Methods

For this study, eSUN PLA-LW (eSUN Industrial Co., Shenzhen, China) was used as the light-weight foaming PLA filament. A conventional PLA filament, eSUN PLA+, was used for comparisons. The cost of the eSUN PLA-LW was 2.1 times the cost of the eSUN PLA + per kilogram.

The 3D printing slicing software Cura (v5.1.0, Ultimaker, Utrecht, Netherlands) was used to prepare prints for an Ender 5 material extrusion 3D printer (Shenzhen Creality 3D Technology Co Ltd, Shenzhen, China) with a 0.4 mm nozzle, a consistent layer height of 0.2 mm, a print speed of 80 mm s^− 1^ and no top or bottom layer used unless otherwise specified. The 3D printing slicing software ideaMaker (v4.2.3, Raise3D, Irvine, USA) was used to prepare prints for a Raise3D Pro 2 material extrusion 3D printer, with a 0.8 mm nozzle, a consistent layer height of 0.4 mm, a print speed of 80 mm s^− 1^, and no top or bottom layer used unless otherwise specified. There were no variations in flow rate or layer height over the duration of the print, for example, for initial layer. The fan was set to a speed of 100% after 3 or 1 layers, on the Ender 5 and Raise3D Pro 2 printers, respectively (corresponding to layer heights of 0.6 and 0.4 mm).

The expansion of the foaming PLA was characterised for a variety of temperatures, using a method similar to that described by filament manufacturer colorFabb [12]. Small hollow test cubes (2 × 2 × 2 cm^2^) were each printed using the eSUN PLA-LW material with a single shell (or outer wall, or perimeter), 1 bottom layer, and no in-fill, at temperatures ranging from 200 to 260 °C (in 10 °C increments, with 260 °C the maximum temperature safely supported by the Ender 5 printer). Test cubes were printed one-per-job in the centre of the print bed, in contrast to the method described by colorFabb [12], where all cubes were printed in one job. Per-model temperature variations are not supported within all slicing software, and print pauses associated with temperature changes increase total print time. Unlike the colorFabb method [12], print speed was not reduced, as the anticipated use of the filament was for 3D printed phantoms, which are already slow to print due to large volumes and high in-fill percentages, and print times would already be extended due to flow rate reductions.

The width of the shell was measured for each side of the cube using calipers. This measurement was exclusive of any unintended imperfections within the print, including Z seam and any minor expansion at base of print (0.1 mm “elephant feet” were observed in this study). An expansion ratio was calculated as a ratio of the measured shell thickness, and the measured shell thickness of an equivalent test cube printed with non-foaming eSUN PLA + at a temperature of 220 °C. The shell thickness for the non-foaming eSUN PLA + printed cube was expected to approximately match the nominal nozzle diameter used.

Subsequently, for each temperature, a test cube was printed using a gyroid in-fill pattern, variable in-fill densities, and an extrusion or flow rate equal to the reciprocal of the calculated expansion ratio. It was expected, that for this flow rate, the smaller volume of PLA-LW material extruded through the nozzle would expand to match the extrusion diameter of non-foaming PLA+. Test cubes were printed at in-fill densities of 20%, 40%, 60%, 80% and 100%, to establish the relationship between in-fill density and radiological properties. The assumption that the reciprocal of the expansion ratio is an appropriate flow rate can be validated by inspection of 100% in-fill prints for under- or over-extrusion, or by printing single shell cubes with variable flow rates, as suggested by colorFabb [12].

The CT number, effective mass density, ρ_eff_, and effective relative electron density, RED_eff_, of the printed test cubes were characterised via CT imaging on a Siemens SOMATOM scanner operating at 120 kVp (Siemens Healthineers AG, Erlangen, Germany) with a slice thickness of 0.5 mm, and a TomoTherapy system operating at 3.5 MVp (Accuray, Sunnyvale, USA), with a slice thickness of 1 mm. The mean, minimum and maximum CT number was sampled from the acquired scans in a central 1 × 1 × 1 cm^3^ region of interest using ImageJ (v1.52p, National Institutes of Health, Bethesda, USA). The ρ_eff_ and RED_eff_ values were interpolated using sampled CT number values in the central 1 × 1 × 1 cm^3^ region of interest of the cubes and characterisation data obtained at commissioning of these imaging systems using a Gammex Model 467 tissue characterization phantom (Gammex Inc, Middleton, USA). To reduce uncertainties in radiological characterisation due to beam hardening effects, all CT imaging was performed with the test cubes surrounded by blocks of water equivalent plastic. This method of characterisation is consistent with the method used by Dancewicz et al. [3].

To facilitate comparison of the new PLA-LW material against the conventional PLA, a set of comparison test cubes were printed using the eSUN PLA + filament at in-fill densities of 20%, 40%, 60%, 80% and 100% using a gyroid in-fill pattern. These calibration cubes were similarly imaged to derive linear relationships between in-fill density and ρ_eff_ and RED_eff_ at both kV and MV energies. The PLA-LW and PLA + cubes were also compared qualitatively, by visual inspection of the acquired images. Reproducibility was assessed by printing of PLA-LW test cubes on the Ender 5 printer at a later date with a new spool of filament, and comparison of kV-derived ρ_eff_ and RED_eff_ values.

## Results

The thickness of the outer contour wall printed with PLA + on the Ender 5 and Raise3D Pro 2 printers were 0.44 ± 0.03 mm and 0.84 ± 0.03 mm respectively, slightly larger than the respective nominal nozzle diameters of 0.4 and 0.8 mm. These PLA + measurements were used as the baseline for calculating the expansion ratios for the PLA-LW printed test cubes are shown in Table 1.

**Table 1:** Expansion ratio and derived flow rate for PLA-LW on Ender 5 and Raise3D Pro 2 printers

Temperature (°C)	Ender 5 with 0.4 mm nozzle		Raise3D Pro 2 with 0.8 mm nozzle	
	Expansion ratio	Flow rate	Expansion ratio	Flow rate
200	1.11 ± 0.07	90%	1.6 ± 0.1	64%
210	1.29 ± 0.08	78%	1.9 ± 0.1	53%
220	1.66 ± 0.08	60%	2.3 ± 0.1	44%
230	2.16 ± 0.12	46%	2.6 ± 0.1	38%
240	2.44 ± 0.11	41%	2.6 ± 0.1	39%
250	2.69 ± 0.11	37%	2.8 ± 0.1	36%
260	2.71 ± 0.08	37%	2.8 ± 0.2	36%

The radiological densities (ρ_eff_ and RED_eff_ for both kV and MV energies) of all test-cubes printed with an in-fill density of 100% are shown for the Ender 5 0.4 mm nozzle and Raise3D Pro 2 0.8 mm nozzle in Tables 2 and 3, respectively.

**Table 2:** Measured effective density, ρ_eff_, and effective relative electron density, RED_eff_, for kV and MV imaging beams for test cubes printed on the Ender 5 printer with 0.4 mm nozzle

Test cube	CT#_kV_ (HU)	ρ_eff,kV_ (g cm^− 3^)	RED_eff,kV_	CT#_MV_ (HU)	ρ_eff,MV_ (g cm^− 3^)	RED_eff,MV_
PLA+, 220 °C	140 ± 22 (98,197)	1.16 ± 0.02 (1.12,1.22)	1.15 ± 0.02 (1.11,1.20)	127 ± 37 (19,251)	1.12 ± 0.04 (1.01,1.25)	1.10 ± 0.04 (0.99,1.23)
PLA-LW, 200 °C	-23 ± 22 (-79,40)	1.00 ± 0.02 (0.94,1.06)	0.98 ± 0.02 (0.93,1.05)	-28 ± 32 (-128,58)	0.96 ± 0.04 (0.85,1.05)	0.94 ± 0.03 (0.84,1.03)
PLA-LW, 210 °C	-152 ± 32 (-225,-85)	0.87 ± 0.03 (0.80,0.94)	0.86 ± 0.03 (0.78,0.92)	-157 ± 29 (-349,-60)	0.82 ± 0.03 (0.62,0.92)	0.81 ± 0.03 (0.61,0.91)
PLA-LW, 220 °C	-376 ± 29 (-463,-326)	0.64 ± 0.03 (0.56,0.69)	0.63 ± 0.03 (0.54,0.68)	-364 ± 34 (446,-263)	0.60 ± 0.04 (0.52,0.71)	0.59 ± 0.04 (0.51,0.70)
PLA-LW, 230 °C	-520 ± 23 (-584,-453)	0.50 ± 0.02 (0.43,0.57)	0.49 ± 0.02 (0.42,0.55)	-488 ± 35 (-591,-407)	0.47 ± 0.04 (0.37,0.56)	0.46 ± 0.04 (0.36,0.55)
PLA-LW, 240 °C	-595 ± 25 (-673,-538)	0.42 ± 0.02 (0.34,0.48)	0.41 ± 0.02 (0.34,0.47)	-563 ± 33 (-655,-484)	0.39 ± 0.04 (0.30,0.48)	0.39 ± 0.03 (0.29,0.47)
PLA-LW, 250 °C	-657 ± 20 (-714,-601)	0.36 ± 0.02 (0.30,0.42)	0.35 ± 0.02 (0.29,0.41)	-636 ± 31 (-723,-556)	0.32 ± 0.03 (0.23,0.40)	0.31 ± 0.03 (0.22,0.39)
PLA-LW, 260 °C	-668 ± 25 (-728,-616)	0.35 ± 0.03 (0.29,0.40)	0.34 ± 0.02 (0.28,0.39)	-669 ± 37 (-741,-570)	0.28 ± 0.04 (0.21,0.39)	0.28 ± 0.04 (0.20,0.38)

**Table 3:** Measured effective density, ρ_eff_, and effective relative electron density, RED_eff_, for kV and MV imaging beams for test cubes printed on the Raise3D Pro 2 printer with 0.8 mm nozzle

Test cube	CT#_kV_ (HU)	ρ_eff,kV_ (g cm^− 3^)	RED_eff,kV_	CT#_MV_ (HU)	ρ_eff,MV_ (g cm^− 3^)	RED_eff,MV_
PLA+, 220 °C	139 ± 37 (32,281)	1.16 ± 0.04 (1.05,1.31)	1.15 ± 0.04 (1.04,1.29)	108 ± 30 (11,212)	1.10 ± 0.03 (1.00,1.21)	1.08 ± 0.03 (0.98,1.19)
PLA-LW, 200 °C	-352 ± 22 (-419,-292)	0.67 ± 0.02 (0.60,0.73)	0.66 ± 0.02 (0.59,0.72)	-336 ± 30 (-427,-240)	0.63 ± 0.03 (0.54,0.73)	0.62 ± 0.03 (0.53,0.72)
PLA-LW, 210 °C	-464 ± 29 (-554,-378)	0.55 ± 0.03 (0.46,0.64)	0.54 ± 0.03 (0.45,0.63)	-460 ± 29 (-551,-383)	0.50 ± 0.03 (0.41,0.58)	0.49 ± 0.03 (0.40,0.57)
PLA-LW, 220 °C	-540 ± 21 (-615,-476)	0.48 ± 0.02 (0.40,0.54)	0.47 ± 0.02 (0.39,0.53)	-514 ± 29 (-586,-426)	0.45 ± 0.03 (0.37,0.54)	0.44 ± 0.03 (0.36,0.53)
PLA-LW, 230 °C	-606 ± 16 (-658,-554)	0.41 ± 0.02 (0.36,0.46)	0.40 ± 0.02 (0.35,0.45)	-527 ± 26 (-590,-456)	0.43 ± 0.03 (0.37,0.51)	0.42 ± 0.03 (0.36,0.50)
PLA-LW, 240 °C	-586 ± 21 (-654,-517)	0.43 ± 0.02 (0.36,0.50)	0.42 ± 0.02 (0.35,0.49)	-560 ± 34 (-645,-476)	0.40 ± 0.04 (0.31,0.49)	0.39 ± 0.04 (0.30,0.48)
PLA-LW, 250 °C	-612 ± 17 (-649,-546)	0.40 ± 0.02 (0.37,0.47)	0.40 ± 0.02 (0.36,0.46)	-592 ± 35 (-670,-482)	0.36 ± 0.04 (0.28,0.48)	0.36 ± 0.04 (0.27,0.47)
PLA-LW, 260 °C	-609 ± 17 (-658,-565)	0.41 ± 0.02 (0.36,0.45)	0.40 ± 0.02 (0.35,0.44)	-578 ± 36 (-659,-502)	0.38 ± 0.04 (0.29,0.46)	0.37 ± 0.04 (0.29,0.45)

Effective mass and electron densities of 0.28 were achievable with 100% in-fill densities, featuring only microscopic air gaps.

**Fig. 1:**
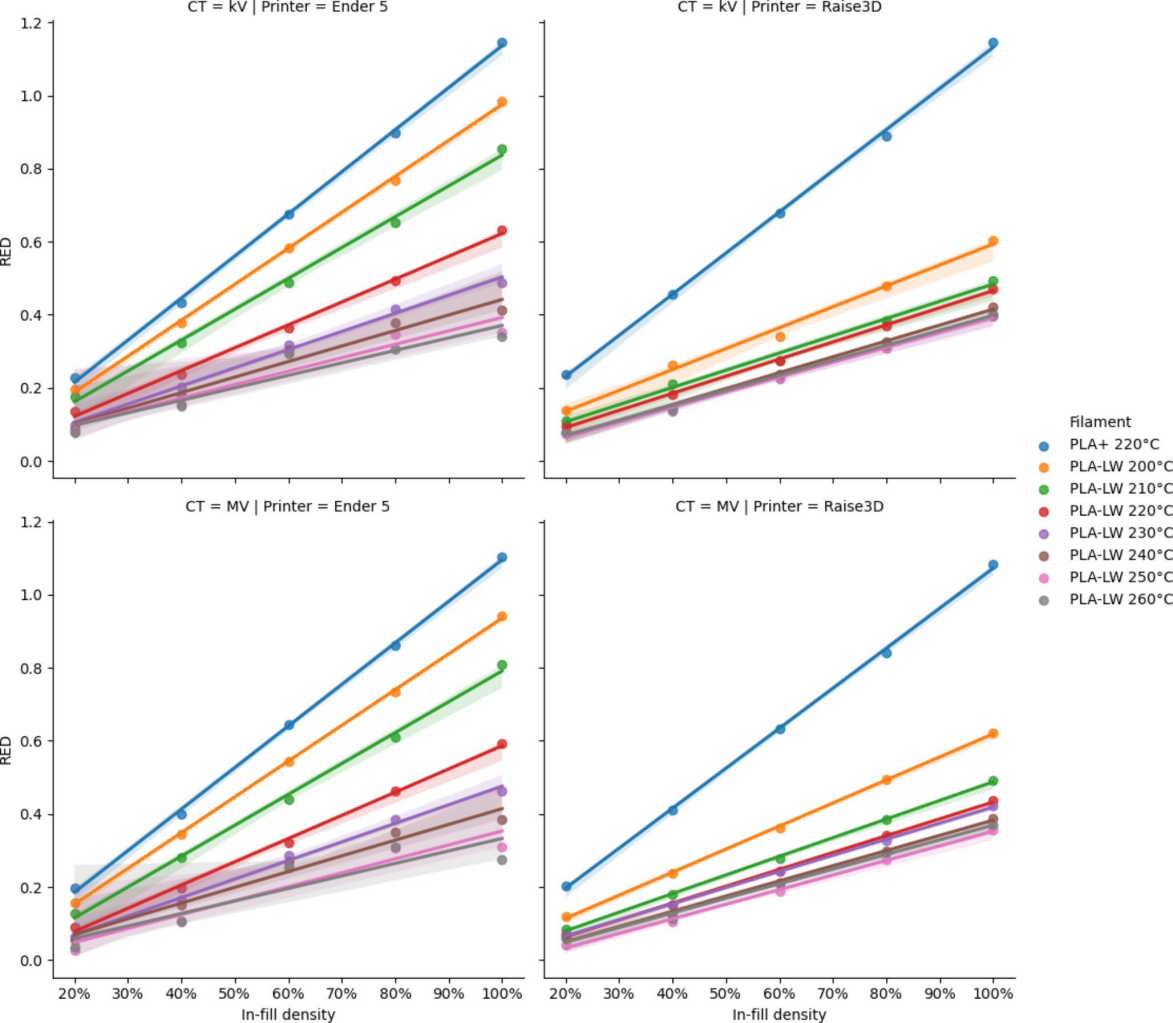
Relationship between in-fill density and RED for test cubes printed on the Ender 5 printer with 0.4 mm nozzle and Raise 3D Pro 2 printer with 0.8 mm nozzle

The relationship between in-fill density and kV and MV CT-derived RED across both printers for different temperatures is shown in Fig. 1.

The kV-derived radiological densities were reasonably reproducible across initial and repeated printing of PLA-LW test cubes, with differences exceeding 0.03 g cm^− 3^ observed for only 5 (of 35) combinations of temperature, flow-rate and in-fill density. These differences were observed at lower temperatures (≤ 230 °C) and may relate to environmental conditions during printing or storage of filament, or variations in filament itself.

**Fig. 2:**
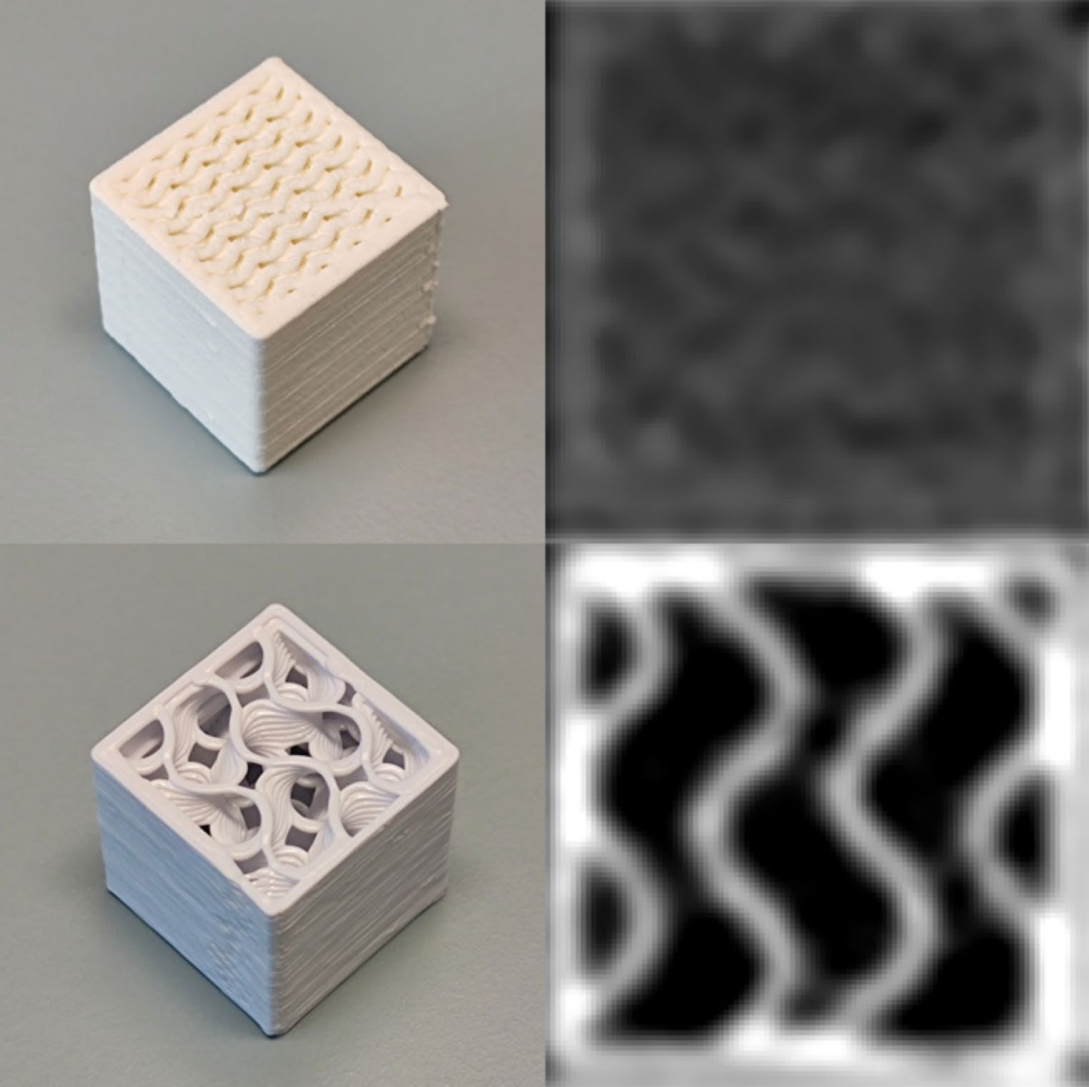
Photographs (left) and central CT slices (right) of test cubes with equivalent RED of 0.24 printed with Raise3D Pro 2 0.8 mm nozzle using PLA-LW at 260 °C at 60% in-fill and a flow of 36% (top) and PLA + at 20% in-fill (bottom), shown with identical window settings (width: 1000, level: -500)

The results demonstrate that PLA-LW supports the printing of lower density tissue substitutes with at greater in-fill densities, resulting in smaller air gaps. This is illustrated in Fig. 2.

## Discussion

The effective density achievable at 100% in-fill was 0.28 g cm^− 3^, approximately 25% of conventional PLA at 100% in-fill, and close to 50% of low-density Woodfill composite, reported as 0.53 by Dancewicz et al. [3].

The radiological characteristics in both kV and MV imaging beams were consistent, with differences between effective densities similar in magnitude to sampled standard deviations. This suggested there is no significant quantity of high-Z ingredient with a radiopacifying effect at lower energies present in the material. This consistency allows the PLA-LW material to be used as a lung-equivalent material in dosimetry studies, for which the characteristics of low-infill PLA have been previously described [4, 13].

There was a substantial difference in expansion between the two printers, which may be due to differences in hot-end design, or the increased thermal mass of the material extruded through the larger nozzle diameter. If the extrusion maintains a temperature at which the chemical foaming agent can decompose for a longer amount of time, this will result in increased volume expansion. The thermal mass effect may also explain why there were minimal gains (in terms of reduction of printed density) with increasing temperature beyond 240 °C on the Raise 3D Pro 2 printer.

There was a minimal increase in effective densities for the PLA-LW material when printed at 230 °C or higher for in-fill densities between 60% and 100% on the Ender 5 printer with the 0.4 mm nozzle, which was not observed for the test cubes printed at lower temperatures or printed on the Raise 3D Pro 2 printer with larger nozzle. Measurements of the mass of these test cubes indicated that the similarity in density was a physical one (i.e., not an imaging or sampling error). This may be due to a printing issue, such as a higher flow rate than expected resulting from the higher temperatures, or a limitation in the method used to optimise flow rate setting.

**Fig. 3:**
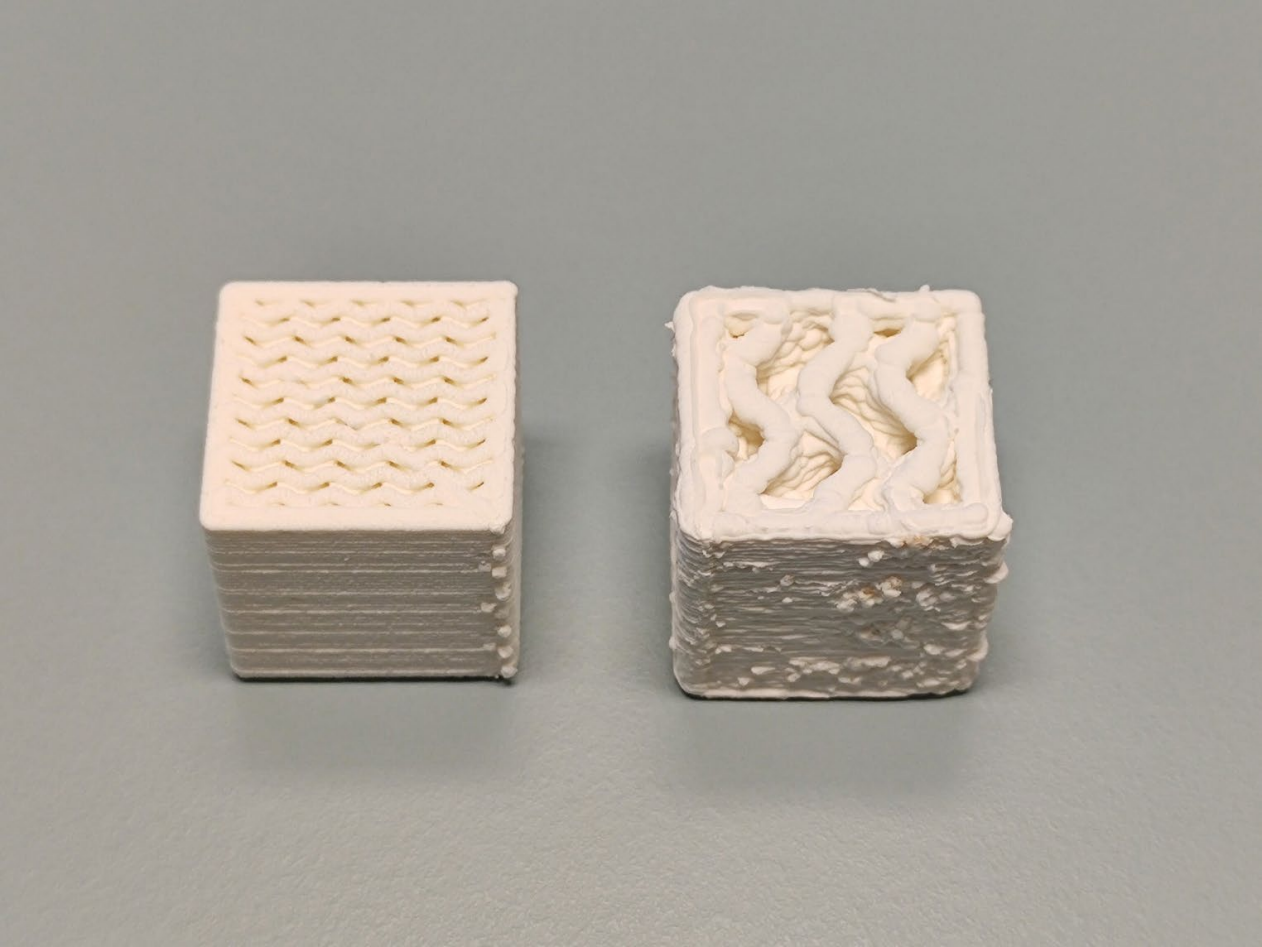
Test cubes with equivalent relative electron density printed with Raise3D Pro 2 0.8 mm nozzle, with 60% in-fill and 36% flow rate (left) and 20% in-fill density and 100% flow rate (right)

As an alternative to the approach described here, the PLA-LW in-fill density could be reduced, instead of the flow rate. This approach could allow elimination of air gaps between extrusion lines (with gaps being filled by the foaming PLA). However, this approach has limitations: the external dimensions of the printed objects would be larger than desired, requiring larger tolerances to be used where parts are intended to be fit together, and over-extrusion at layer transition seams resulting from higher temperatures may introduce print defects (see Fig. 3).

While the PLA-LW material used in this study was approximately 2.1 times the cost of PLA + filament, when printing lung density materials it would be a more cost-efficient material, given the 2.8× reduction in flow rate seen at high temperatures (meaning 1 kg of PLA-LW would produce 2.8x extrusion length of 1 kg of non-foaming PLA).

One limitation in the use of the PLA-LW filament is that it is only able to achieve densities similar to water at lower printing temperatures, and so the fabrication of inhomogeneous phantoms containing lung- and soft- tissue mimicking media would either require printing of components separately and subsequent assembly, the use of a system supporting two or more filaments quasi-simultaneously [7], or the modulation of printing temperature, which would increase print time due to pauses for heating and cooling, and could result in oozing or additional stress on the hot-end.

The use of an increased in-fill density for PLA-LW, compared to non-foaming PLA, would increase travel distance of the print head and result in longer prints.

The high printing temperatures seen in this study should be used with caution, due to the risk of degradation of any polytetrafluoroethylene (PTFE) within the hot end of the printer. For example, for the Raise 3D Pro 2 used in this study, there is minimal benefit to printing at a temperature above 240 °C, so this temperature should not be exceeded. Greater volume expansion can be achieved at lower temperatures using a larger nozzle, which also facilitates increased printing times (due to increased layer height).

Based on the variations observed between the two printers, and the potential for filament batch variations [5], it is recommended that calibration cubes to characterise the relationship between in-fill density, temperature and radiological properties should be printed on other systems and analysed, as described in this study, before use in the fabrication of phantoms. For some printers, depending on printer bed height calibration or print surface, modification of initial layer flow rate, layer height or fan settings may be required for adhesion.

## Conclusion

This study described a method for optimising printer parameters for a light-weight foaming PLA in order to achieve low mass- and electron- densities with smaller air gaps than seen for conventional PLA filaments. This material and technique can be used as a cost-effective method to achieve lung-equivalency in 3D printed phantoms for use in imaging and dosimetry.
